# Skills in handling Turbuhaler, Diskus in the west of China

**DOI:** 10.1186/s12890-023-02765-1

**Published:** 2023-11-17

**Authors:** Wei Wei, Dong Wang, Weiting Liu, Hui Du, Zhiye Zhang, Shengying Che, Rui Ding, YanBiao Yang

**Affiliations:** 1 Department of Pharmacy, Lanzhou First People’s Hospital, Lanzhou, China; 2 Respiratory and Critical Care Department of Lanzhou First People’s Hospital, Lanzhou, China

**Keywords:** Inhaler, Inhalation technique, Pharmacist intervention, Quantitative evaluation

## Abstract

**Objective:**

The purpose of this study was to evaluate the inhaler skills of patients with asthma and chronic obstructive pulmonary disease in a hospital in western China after receiving one medication education by pharmacists and the factors related to these skills.

**Methods:**

We included 96 subjects using Turbuhaler and 74 subjects using Diskus in a hospital in western China. They were educated once by pharmacists before medication, and then their skills of operating these inhalers were visually evaluated the next time they were used. Using the seven-step inhalation administration method designed by AnnaMurphy, a clinical pharmacist at GLENFIELD Hospital in the UK, the inhaler use technique score scale was established and scored in turn. The age, sex, time of first illness, smoking status, education level and type of health insurance purchased by each patient were recorded to assess their relationship with overall inhaler skills.

**Results:**

19.8% of the subjects who used Turbuhaler could not use it correctly, and 43.2% of the subjects who used Diskus could not use it correctly. The step with the highest error rate with Turbuhaler and Diskus is to "exhale slowly to residual volume". Chi-square test was carried out for each step of the operation of the two kinds of inhalers, and it was found that there was a significant difference in the operation accuracy of the two kinds of inhalers in the first, third and eighth steps. In univariate analysis, advanced age, female and low educational level were related to the lack of inhaler technology, but in multivariate analysis, only low educational level was a significant independent risk factor.

**Conclusion:**

Among the patients with asthma and chronic obstructive pulmonary disease in western China, some patients have good inhaler operation skills, but there are still many patients who can not use inhalers correctly, and the lower education level is significantly related to the incorrect use of inhalers.

**Supplementary Information:**

The online version contains supplementary material available at 10.1186/s12890-023-02765-1.

## Introduction

Inhalation therapy is very important in the treatment of Asthma and chronic obstructive pulmonary disease (COPD). The Global Asthma Initiative (GINA) guidelines recommend regular use of inhaled corticosteroids with or without long-acting beta 2 agonists, and inhaled rapid acting beta 2 agonists as needed to relieve symptoms [1]. The Global Initiative for Chronic Obstructive Pulmonary Disease (GOLD) guidelines recommend inhaled therapy for stable chronic obstructive pulmonary disease is also critical [2]. The main advantage of inhalant therapy is that it can reach the lungs directly with a small dose, the side effect is less than oral treatment [3]. However, the good therapeutic effect of inhaled drugs is closely related to the patient’s ability to use the inhaler correctly [4]. Previous studies have shown that persistent use of inhaled drugs is associated with different inhalation devices [5], and related studies have pointed out that self-reported compliance measured by MARS-5 is inaccurate in determining non-compliant inhaled drugs in COPD patients [6]. Therefore, a study used the seven-step inhalation administration method designed by AnnaMurphy, a clinical pharmacist at GLENFIELD Hospital in the UK [7], to evaluate patients’ inhalation and use compliance. A study in Korea found that most of the inhaler skills were excellent, while the older age and lack of previous guidance on operating the inhaler were related to the lack of skills [8]. A joint study in Australia and Jordan showed that most patients in both regions could not keep their inhalers upright and could not exhale to residual volume, exhale away from mouthpiece [9]. It can be seen that in many countries and regions, there are patients who can not use inhalers correctly.

Most of the current studies are carried out in economically developed areas, and the economic level of western China is relatively backward, so that the education level of patients is low. According to the instructions, inhalers can not be used correctly. The main purpose of this study is to investigate the inhaler skills of patients in the author’s hospital, in order to clarify the basic situation of inhaler use of patients in western China, and lay a good foundation for better service for patients.

## Materials and methods

### Subjects

We enrolled 170 patients who used inhalers for respiratory diseases (bronchial asthma, chronic obstructive pulmonary disease), Of these, 96 were Turbuhaler users and 74 Diskus users. The reason for choosing these two kinds of equipment for research is that they are currently the most commonly used devices for patients with bronchial asthma and chronic obstructive pulmonary disease in our hospital, and the operation of the two devices is different. This study was approved by the hospital ethics committee and the informed written consent of the patients. This study included patients who used Turbuhaler and Diskus during hospitalization in respiratory and critical care department of our hospital in the past two months, and no exclusion standard was set.

### Evaluate the appropriateness of different devices

Before using Turbuhaler and Diskus, the pharmacist first instructs the user to use the drug to ensure that the patient can use the device correctly. The pharmacists who guide the use of drugs are pharmacists who have received standardized training and have skillfully mastered the use methods and matters needing attention of different inhalers.Visual assessment of patients’ use of Turbuhaler and Diskus was performed the next day, excluding opinions or other influences that might come from doctors or other medical staff. Some patients have used the two inhalers included in this study, but they have received professional guidance from pharmacists, so they will not affect the results of this study. Based on the seven-step inhalation method designed by AnnaMurphy, a clinical pharmacist at GLENFIELD Hospital in the UK, the inhalation technology assessment of Turbuhaler and Diskus includes the following nine aspects:1) open the, vice correctly;2) load and prime device;3) exhale to residual volume slowly;4) mouth holding device;5) lift your head as you inhale to open the airway;6) hold breath for 5–10 s;7) rinse mouth;8) know how to reuse drugs and how to reuse them;9) clean the device and the method is correct. In this study, an inhaler training means that pharmacists explain and demonstrate to patients according to the seven-step inhalation process, and patients gradually explain and demonstrate each step for pharmacists after learning, so as to skillfully use the corresponding inhalers. In addition, according to the degree of completion of the defined steps, the overall technology of the inhaler is divided into three levels, including good, adequate and inadequate. If the subjects were able to perform each step correctly, they were rated as good. The subjects were able to correctly complete the key steps, including " open the device correctly “, " load and prime device " and " exhale to residual volume slowly “, but were rated adequate when they did not complete all the other steps. If any of the key steps are not completed correctly, they will be assessed as inadequate.

### Collect information

Other demographic parameters of the subjects need to be collected, including age, sex, duration of the disease, smoking status, education level, and health insurance (employee health insurance and resident health insurance).

### Statistical analysis

Demographic data were compared according to the types of DPI that subjects often used for asthma control (Turbuhaler and Diskus), using Mann-Whitney-U test (continuous variables) and ${x}^{2}$test (classified variables). A comparison was also made between the good or appropriate group and the unqualified group of each type of inhaler. At the same time, the operation of each step is compared.In the univariate analysis, the Logistic regression model was established by taking the demographic variables related to the lack of inhaler operation technology as independent variables and the lack of inhaler operation technology as dependent variables. The forward selection method is used to eliminate the multiple collinearity of each variable. Statistical software package (SPSS26.0) was used for statistical analysis. *P* < 0.05 was considered to be statistically significant.

## Results

### Demographic and clinical characteristics

All subjects were treated in a hospital in western China. Table 1 shows the demographic characteristics of patients using Turbuhaler and Diskus. We can see that there is no difference in age, sex, length of time of illness, education and health insurance between the two groups. With regard to smoking, there was no difference in the number of current smoker, former smoker and never smoker did not differ between the two groups.

**Table 1:** Demographic characteristics of patients according to the type of DPI(Turbuhaler vs. Diskus) used for disease control

	The type of DPI		*P* value
	Turbuhale users(n = 96)	Diskus users(n = 74)	
Age(mean ± SD)	59.33 ± 13.77	59.82 ± 15.22	0.714
Male sex(%)	46(47.92)	38(51.35)	0.757
Duration of illness(mean ± SD)	7.2 ± 9.4	8.5 ± 10.1	0.709
Smoking status			
Current smoker(%)	13(13.54)	10(13.51)	1.000
Former smoker(%)	11(11.46)	14(18.92)	0.195
Never smoker(%)	72(75)	50(67.57)	0.307
Highest grade completed in school			
College or further (%)	30(31.25)	28(37.84)	0.416
High school (%)	30(31.25)	22(29.73)	0.868
Middle school or less (%)	36(37.50)	24(32.43)	0.521
basic medical insurance system for urban workers and residents	46(47.92)	39(52.70)	0.643

### Technique for each step in handling the Turbuhaler and Diskus

**Fig. 1:**
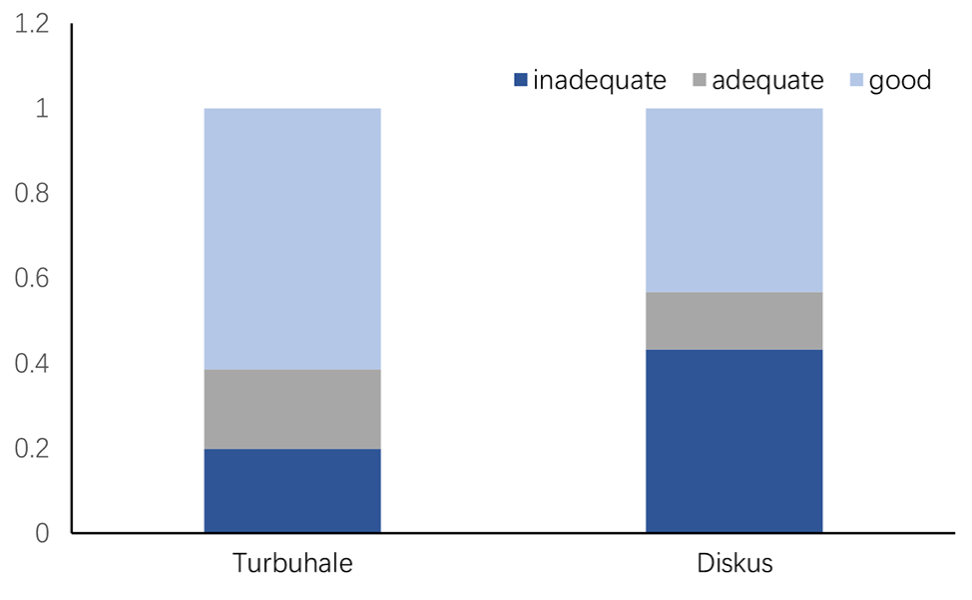
Technical evaluation of different inhalers. The part of the bar chart shows the percentage of subjects whose overall skills are rated as good, qualified or unqualified

**Fig. 2:**
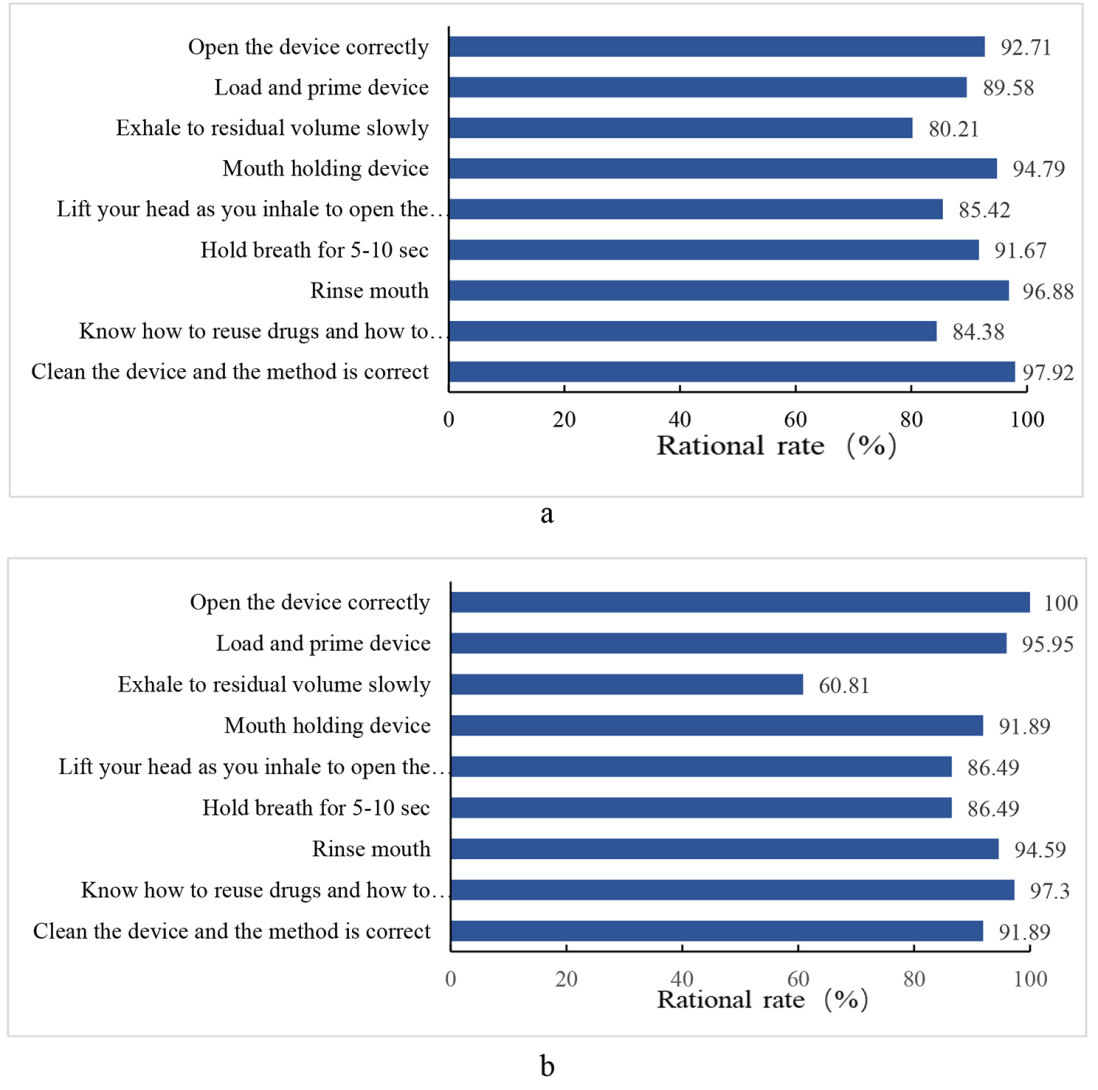
The percentage of subjects who completed each step with each inhaler. (**a**) Turbuhaler; (**b**) Diskus. The bar represents the proportion of subjects who have completed the relevant steps

The overall assessment of inhaler use technology showed that 61.4% of the subjects who used Turbuhaler were technically good, 18.8% were adequate, and 19.8% were inadequate, while 43.3% of Diskus subjects were technically good, 13.5% were adequate, and 43.2% were inadequate (Fig. 1). In terms of the proportion of subjects who completed each step, the proportion of subjects using Turbuhaler to complete each step was 92.71%, 89.58%, 80.21%, 94.79%, 85.42%, 91.67%, 96.88%, 84.38%, 97.92% respectively (Fig. 2a). The percentages of subjects using Diskus to complete each step were 100%, 95.95%, 60.81%, 91.89%, 86.49%, 86.49%, 94.59%, 97.3%, 91.89%, respectively (Fig. 2b).

Comparing each operation of different DPI, it was found that there were statistical differences in three operation steps between two different inhalers, they are “Open the device correctly” and “Exhale to residual volume slowly” and “Lift your head as you inhale to open the airway”. (*P*<0.05) There is no difference in other operating steps between the two inhalers. (Table 2)

**Table 2:** Comparison of each operation of different DPI

	operation sequence	Turbuhale		Diskus		${x}^{2}$	*P*
		operate successfully	failed	operate successfully	failed		
1	Open the device correctly	89	7	74	0	5.628	**0.019**
2	Load and prime device	86	10	71	3	2.395	0.152
3	Exhale to residual volume slowly	77	19	45	29	7.760	**0.006**
4	Mouth holding device	91	5	68	6	0.581	0.536
5	Lift your head as you inhale to open the airway	82	14	64	10	0.039	0.512
6	Hold breath for 5–10 s	88	8	64	10	1.184	0.320
7	Rinse mouth	93	3	70	4	0.550	0.470
8	Know how to reuse drugs and how to reuse them	81	15	72	2	7.753	**0.008**
9	Clean the device and the method is correct	94	2	68	6	3.382	0.080

### Factors associated with inadequate inhaler techniques

We evaluated the factors related to the inadequate use of technology in Turbuhaler and Diskus. A comparison was made between subjects with good or adequate use of inhalation devices and subjects with poor use of inhalers (Table 3). The results show that the older age is related to the incorrect use of Turbuhaler and Diskus. Men are also associated with incorrect use of Turbuhaler. And the lower level of education is also related to the incorrect use of Turbuhaler and Diskus. Taking the poor use of Turbuhaler technology as dependent variables, age, gender and education level as independent variables, a Logistic regression model with imperfect overall processing technology was established. It was found that age, sex and education level were not independent risk factors of Turbuhaler technology use. Taking the poor use of technology of Diskus as dependent variables and age and education level as independent variables, the Logistic regression model was established. The results showed that low level of education (OR:6.58,95%CI:2.115–20.468) was an independent risk factor for insufficient operating skills, while age were excluded during for-ward selection. (Table 4)

**Table 3:** Factors associated with inadequate inhaler techniques

	Turbuhale			Diskus		
	Good or adequate(n = 77)	inadequate(n = 19)	*P* value	Good or adequate(n = 42)	inadequate(n = 32)	*P* value
Age(mean ± SD)	**57.39 ± 13.38**	**67.21 ± 12.80**	**0.008**	**56.02 ± 16.64**	**64.81 ± 11.58**	**0.016**
Male sex(%)	**41(53.2)**	**5(26.3)**	**0.043**	19(45.2)	19(59.4)	0.250
Duration of illness(mean ± SD)	5.83 ± 6.31	12.58 ± 16.10	0.234	8.10 ± 10.02	9.06 ± 10.37	0.549
Smoking status						
Current smoker(%)	12(92.3)	1(7.7)	0.454	4(40)	6(60)	0.313
Former smoker(%)	9(81.8)	2(18.2)	1.000	7(50)	7(50)	0.765
Never smoker(%)	56(77.8)	16(22.2)	0.385	31(62)	19(38)	0.217
Highest grade completed in school						
College or further (%)	27(35.1)	3(15.8)	0.166	20(47.6)	8(25)	0.056
High school (%)	26(33.8)	4(21.1)	0.409	16(38.1)	6(18.8)	0.080
Middle school or less (%)	**24(31.2)**	**12(63.2)**	**0.016**	**6(14.3)**	**18(56.3)**	**0.000**
Basic medical insurance system for urban workers(%)	40(51.9)	6(31.6)	0.130	19(45.2)	13(40.6)	0.100

**Table 4:** Adjusted oddsratiosfor inadequate inhaler technique

	Odd Ratios(95%CI)	*P* value
**Turbuhale**		
Age	2.915(0.853–9.964)	0.088
Sex	0.310(0.092–1.048)	0.059
Middle school or less	2.304(0.723–7.346)	0.158
**Diskus**		
Age	2.166(0.759–6.184)	0.149
Middle school or less	6.580(2.115–20.468)	**0.001**

## Discussion

The subjects in this study were able to correctly handle most of the procedures of Turbuhaler and Diskus. The three procedures with the highest success rate for subjects using Turbuhaler were “Clean the device and the method is correct”, “Rinse mouth” and “Mouth holding device” respectively, and those using Diskus were “Open the device correctly”, “Know how to reuse drugs and how to reuse them” and “Load and prime device” respectively. The most common mistake of the two inhalers is step “Exhale to residual volume slowly”,

When using Turbuhale and Diskus, there are significant differences in the accuracy of three operating steps. Compared with the use of Turbuhale, the success rate of the first and eighth steps of Diskus is significantly higher, which may be related to the tedious operation of Turbuhale in the first step. According to the instructions of Turbuhale, Turbuhale needs to initialize the operation in the first use, and it needs to hold the red handle and keep the middle part, and rotate to the end in a certain direction. Then rotate in the opposite direction to the end, the more complex operation makes it possible for patients to have a low correct rate of operation, so pharmacists or physicians should pay special attention to the first step of the operation when guiding patients to use Turbuhale. The result of this study is consistent with the previous results. [10].

According to relevant literature reports, age is an important determinant of correct inspiratory technology [8, 11]. The results of this study showed that subjects with inadequate treatment techniques when dealing with Turbuhaler or Diskus were significantly older than patients with adequate treatment techniques, but there was no correlation between older age and inadequate management of inhalers in multivariate analysis, which was inconsistent with previous studies.

The results of this study showed that in terms of gender, the inhalation technique of men using Turbuhale was worse than that of women, while previous studies showed that women had worse inhalation techniques than men [12], and other studies showed that there was no significant difference in the use of Turbuhale between men and women [8]. This contradicts the results of previous studies. However, the results of multivariate analysis showed that gender was not a factor affecting the use of Turbuhale.

In terms of the length of medical history, previous studies have shown that patients with a long course of disease lack regular follow-up and are unable to maintain correct inhalation techniques. The researchers analyzed that patients with a long course of disease had to replace different inhalation devices because of their illness, which may cause confusion in usage. In addition, most of the patients with long course of disease are in serious condition, and their lung function is poor and can not inhale drugs effectively. Inadequate inhalation technology may further aggravate the disease and form a vicious circle [13]. This study shows that the correct use of the two inhalers is not related to the length of the medical history, which may be related to the study design, which may affect this factor by giving medication guidance to patients again before recording inhalant use.

This study showed that smoking, having smoked and never smoking had no correlation with the correct use of inhalers. This is consistent with the results of previous studies [8].

The results of previous studies show that the education level of patients is significantly related to the correctness of inhaler use. most of the patients with low educational background have poor understanding ability and low cognitive ability, so they can not master the correct use of inhaler [14] This is consistent with the results of this study. The results of this study showed that a lower level of education was significantly associated with incorrect use of inhalers, regardless of Turbuhale or Diskus. The results of establishing Logistic regression model show that only the incorrect use of Diskus is related to the lower level of education, but this is not consistent with the existing experience, because in the course of daily operation, Turbuhale is more complex than Diskus, which may be related to the multicollinearity in the process of Logistic regression. [15] Multicollinearity means that the model estimation is distorted or difficult to estimate accurately because of the exact correlation or high correlation between the explanatory variables in the linear regression model.

This study is only a statistical analysis of the included subjects after a drug education, the study found that the operation error rate of patients in the first and eighth steps of using Turbuhale was higher than that of Diskus. Univariate test found that the level of use of the two inhalers included in the study was related to age, gender and education level, but only the correct use rate of Diskus was related to education level after logical regression. The main deficiency of this study is that the follow-up time is short, the influence of inhaler counter on compliance is not analyzed in detail, the relationship between inhaler and correct use of inhaler is not analyzed by using compliance analysis scale [6], and the equipment for measuring peak inspiratory flow rate is not introduced, so it is impossible to evaluate the use of inhaled drugs, so the standardized training method of inhaler is proposed in the next step. The relationship between inhalation technology and the degree of lung function damage, disease severity, drug treatment effect, patient satisfaction with the device and other factors were evaluated [16].

In conclusion, The correct rate of dry powder atomization inhalation in some patients with chronic respiratory diseases in western China is low. Pharmacists are required to strengthen their return visits after standardizing drug use education, or it is possible to solve this problem by improving inhaler equipment [17].

### Electronic supplementary material

Below is the link to the electronic supplementary material.

Supplementary Material 1

## Data Availability

The raw data supporting the conclusions of this article will be made available by the authors, without undue reservation. For data acquisition, please contact Wei Wei.
